# Quantification of pro-inflammatory cytokines and osteoclastogenesis markers in successful and failed orthodontic mini-implants

**DOI:** 10.1590/1678-7757-2018-0476

**Published:** 2019-10-07

**Authors:** Marcela Cristina Damião Andrucioli, Mírian Aiko Nakane Matsumoto, Sandra Yasuyo Fukada, Maria Conceição Pereira Saraiva, Ana Zilda Nazar Bergamo, Fábio Lourenço Romano, Raquel Assed Bezerra da Silva, Lea Assed Bezerra da Silva, Paulo Nelson-Filho

**Affiliations:** 1 Universidade de São Paulo Faculdade de Odontologia de Ribeirão Preto Departamento de Clínica Infantil Ribeirão PretoSão Paulo Brasil Universidade de São Paulo, Faculdade de Odontologia de Ribeirão Preto, Departamento de Clínica Infantil, Ribeirão Preto, São Paulo, Brasil.; 2 Universidade de São Paulo Faculdade de Ciências Famacêuticas de Ribeirão Preto Departamento de Física e Química Ribeirão PretoSão Paulo Brasil Universidade de São Paulo, Faculdade de Ciências Famacêuticas de Ribeirão Preto, Departamento de Física e Química, Ribeirão Preto, São Paulo, Brasil.

**Keywords:** Orthodontic anchorage procedures, Cytokines, RANK, RANK ligand, Osteoprotegerin

## Abstract

**Objectives:**

Miniscrew has been frequently used, considering that anchorage control is a critical point in orthodontic treatment, and its failure, the main adverse problem. Using two groups of stable (successful) and unstable (failed) mini-implants, this *in vivo* study aimed to quantify proinflammatory cytokines IL-1 α, IL-6, IL-17, and TNF-α and osteoclastogenesis marker RANK, RANKL, and OPG in gingival tissue, using the real-time polymerase chain reaction technique.

**Methodology:**

Thirteen patients of both sexes (11-49 years old) under orthodontic treatment were selected, obtaining 11 successful and 7 failed mini-implants. The mini-implants were placed and removed by the same surgeon, in both jaws. The mean time of permanence in the mouth was 29.4 months for successful and 7.6 months for failed mini-implants. At removal time, peri-mini-implant gingival tissue samples were collected and processed for quantification of the proinflammatory cytokines and osteoclastogenesis markers. Nonparametric Wilcoxon rank-sum test considering the clusters and Kruskal-Wallis test were used for statistical analysis (α=0.05).

**Results:**

No significant difference (p>0.05) was observed between the groups for either quantification of cytokines or osteoclastogenesis markers, except for IL-6 (p<0.05).

**Conclusions:**

It may be concluded that the expression of IL-1α, IL-17, TNF-α, RANK, RANKL, and OPG in peri-implant gingival tissue were not determinant for mini-implant stability loss, but the higher IL-6 expression could be associated with mini-implant failure.

## Introduction

Mini-implants have been widely used in orthodontics^1^,^2^ with a high clinical success rate.^3^ Even so, loss of devices may still occur in some cases. The reasons for failures are not entirely clear. According to a recent revision,^4^ failure was associated with initial loading, inadequate hygiene, brushing, or thrusting in the area, and persistent inflammation. Moreover, inflammation is frequently mentioned as one of the factors involved in implant loss.^3^,^5^,^6^

The installation of dental implants promotes the activation of molecular mechanisms involved in bone remodeling for osteointegration and can also trigger a cascade of inflammatory reactions by the stimulus of cytokine and chemokine production, contributing to the establishment of a unique biochemical environment.^7^–^9^

Moreover, inflammatory and immune response increases in peri-implant periodontal tissues with increased microbial colonization of implants after installation in the oral cavity, resulting in greater production of proinflammatory cytokines.^7^,^10^ The expression of these proinflammatory cytokines and osteoclastogenesis-related factors plays an important role in the development and severity of peri-implantitis, a major cause of dental implant loss.^11^–^13^

A balance between pro-inflammatory and anti-inflammatory cytokines regulates immune response. These mediators are also key regulating factors of osteoclast and osteoblast differentiation and activation, which modulate osteoclastogenesis and maintain bone homeostasis, especially in response to aggressive agents.^7^,^10^

IL-1α and IL-6 have a trigger in osteoclast differentiation and proliferation, improving bone loss. TNF-α is released by lymphocytes, fibroblasts, leukocytes, and epithelial cells of the gingival tissue and has a key role in the inflammatory process by inducing the over-expression of the nuclear factor κB (Receptor Activator of Nuclear Factor Kappa-Β Ligand)^14^,^15^ and, consequently, bone resorption. IL-17 is a proinflammatory cytokine, and its receptors are expressed by osteoclasts, osteoblasts, synoviocytes, and chondrocytes cells. IL-17 increases *RANKL* expression in osteoblasts cells, as well as TNF-α and *IL-1* production in synovial macrophages.^16^–^18^ RANK/RANKL/OPG system is a key signaling molecules that facilitate cross-talk between osteoblasts and osteoclasts. OPG inhibits the binding of RANK to RANKL, thus inhibiting osteoclast recruitment, proliferation, and activation. Abnormalities in the balance of *RANK/RANKL/OPG* system increase bone resorption.^19^,^20^

Due to the scarcity of studies, it is not known whether cytokines can cause development of peri-implantitis in orthodontic mini-implants as well. The evaluation of these mediators involved in inflammation is critical to increase the stability of temporary anchorage devices in orthodontics.^8^

Only few studies evaluated the expression of some cytokines - IL-1 β, IL-2, IL-6, and IL-8 – in peri-implant crevicular fluid in response to orthodontic tooth movement,^21^,^22^ but no studies evaluated cytokines and osteoclastogenesis mediators associated with successful and failed mini-implants. Therefore, this study evaluated the gene expression of pro-inflammatory cytokines *IL-1*α, *IL-6, IL-17, TNF-*α and osteoclastogenesis mediators *RANK, RANKL*, and *OPG* in peri-mini-implant gingival tissue samples, using real-time polymerase chain reaction, to verify whether gingival inflammation and bone resorption could be relevant for implant failure.

## Methodology

After approval of the research protocol by the institutional Ethics Committee (Process #19866013.0.0000.5419), the study purposes were fully explained to patients or their legal representatives, who signed a written informed consent form for participation.

Thirteen patients of both sexes, aged between 11 and 49 years, under corrective orthodontic treatment with fixed appliances at the Orthodontics Clinic and indication for orthodontic anchorage with mini-implants were enrolled in this study for 12 months, after clinical interview and examination.

As previously described by Andrucioli, et al.^23^ (2018), participants had good general and oral health, were nonsmokers and had not used antibiotics or anti-inflammatory drugs within 3 months before mini-implant removal. Two groups of mini-implants were obtained: 11 well-fixed mini-implants (successful), removed after completion of orthodontic mechanics or at the end of the treatment; and 7 unstable mini-implants, which became loose before the production of the desired tooth movement, thus being removed earlier (failed).

Mini-implants (1.6 mm diameter x 7.0 or 9.0 mm long; Neodent; Curitiba, PR, Brazil) were placed in both jaw sides. All mini-implants were placed and removed by the same experienced surgeon using the same surgical technique and without contact with adjacent tooth roots. All devices presented primary stability immediately after placement. All patients received the same postsurgical instructions to clean the peri-implant area with a soft bristle brush during tooth brushing and rinse the mouth with an antiseptic solution once a day during the use of the mini-implant. The mean time of permanence in the mouth was 29.4 months for successful mini-implants and 7.6 months for failed ones.

At mini-implant removal, 1 mm of peri-implant gingival tissue was also removed using a scalpel blade and placed individually in RNAse-free 2.0 mL microcentrifuge tubes (Eppendorf AG, Hamburg, Germany) containing 200 µL of Trizol (Gibco BRL, Life Technologies, Rockville, MD, USA). The tubes were frozen at −80°C until processing by RT-PCR.

### RNA extraction and cDNA synthesis by quantitative real-time PCR analysis

mRNA expression was evaluated for quantification of pro-inflammatory cytokines (*IL-1*α, *IL-6, IL-17, and TNF-*α) and osteoclastogenesis markers (*RANK, RANKL*, and *OPG*). The samples were defrosted, ground in a polytron (Ultra Turrax, Ika-Werke, Staufen, BW, Germany), homogenized by agitation in a shaker (Mixtron; Toptronix, São Paulo, SP, Brazil) for 30 s for desorption of the material adhered to the mini-implant surfaces and maintained at room temperature for 5 minutes. Total RNA extraction was performed using an extraction kit (Promega Corp., Madison, WI, USA) following the manufacturer's instructions. Aliquots of 2 µL were used to determine RNA concentration (µg/µL) in each sample using a full-spectrum, UV-Vis spectrophotometer (Nanodrop 2000; Thermo Fisher Scientific Inc., Waltham, MA, USA). After RNA extraction, complementary DNA (cDNA) was synthesized using 1 mg of RNA and the Improm II Reverse Transcription System kit (Promega Corp.).

Quantitative real-time PCR analysis of mRNA expression was performed on a StepOnePlus™ RT-PCR system (Applied Biosystems^®^, Foster City, CA, USA) using SYBR-Green fluorescence system (Applied Biosystems^®^, Foster City, CA, USA) for quantification of the amplification products. PCR conditions were 10 minutes at 95°C, then 40 cycles of 94°C (1 minute), 58°C (1 minute) and 72°C (2 minutes), followed by a standard denaturation curve. The sequences of primer pairs for the specific amplification of cytokines, osteoclastogenesis-related factors, and reference gene (*GAPDH*) were designed using the IDT primer quest software (Integrated DNA Technologies, Inc., Coralville, IA, USA) and the nucleotide sequence present in the GenBank database (www.ncbi.nlm.nih.gov/Genbank/): *IL-1*α, *IL-6, IL-17*, and *TNF-*α, *RANK, RANKL, OPG*, and *GAPDH*.

PCR conditions for each gene of interest were optimized regarding primer concentration, absence of primer dimer formation and gene amplification efficiency. The integrity of each reaction was confirmed by the presence of a single peak in the melting curve analysis. In each reaction, 300 nM of specific primer, 2.0 µL of cDNA, and SYBR^®^ Select Master Mix (Applied Biosystems, Foster City, CA, USA) were used. The threshold for determination of RT-PCR positivity was established based on negative controls (absence of sample and of reverse transcriptase enzyme).

For mRNA analysis, the relative expression level of the gene of interest was estimated according to the manufacturer's instructions (Applied Biosystems User's Bulletin - P/N 4303859), having *GAPDH* expression in the same sample as a reference, using the cycle threshold (Ct) method. The average of Ct values obtained in duplicate was used to estimate gene expression level, normalized by an internal control (*GAPDH*) and compared with values of the target gene for calculation of the relative increase in gene expression, using the 2^−ΔCt^ formula, according to the manufacturer's instructions. Data were presented as percentage of *GAPDH* expression. *RANKL/OPG* ratio was also estimated.

### Statistical analysis

Comparative analysis of patients, relative to gender and age, and of mini-implants, relative to the mean time of permanence in the mouth, was performed by the test of difference of means for continuous variables and test of difference of proportions (Wald test) for categorical variables, considering the individuals as conglomerates.

All results were analyzed considering clusters within individuals. Comparisons of all cytokine and osteoclastogenesis mediators, between successful and failed mini-implants, were performed by the nonparametric Wilcoxon rank-sum test considering the clusters^24^. All analysis were performed using the SAS (Statistical Analysis System) software for Windows version 9.3 (SAS Institute, Inc., Cary, NC, USA). Significance level was set at 5%.

## Results

Peri-implant gingival tissue samples of 18 mini-implants (7 failed and 11 successful) were obtained from 13 patients. Descriptive data analysis showed no statistically significant difference between the groups regarding age and gender. The only significant difference (p=0.0116) was the meantime of permanence in the mouth, as the successful mini-implants remained in the mouth for a significantly longer time than failed mini-implants (Table 1).

**Table 1:** Descriptive analysis of data referring to the patients and to the mini-implants of both groups (successful and failed) used for quantification of cytokines and osteoclastogenesis markers

	Successful mini-implants	Failed mini-implants	P value*
	(n=11)	(n=7)	
Sex			
Male	45.5%	14.3%	0.2757
Female	54.5%	85.7%	
Time of permanence in the mouth	29.4 months	7.57 months	0.0116†
(mean; SE**)	(SE =6.8)	(SE =1.9)	
Age	34.0 years	21.7 years	0.0837
(mean; SE)	(SE =5.8)	(SE=4.0)	

* p referring to the Wald test for categorical variables or the difference of means test for continuous variables, considering the dependence of data among the patients

** SE = Standard Error

† Statistically significant difference.

**Figure 1:**
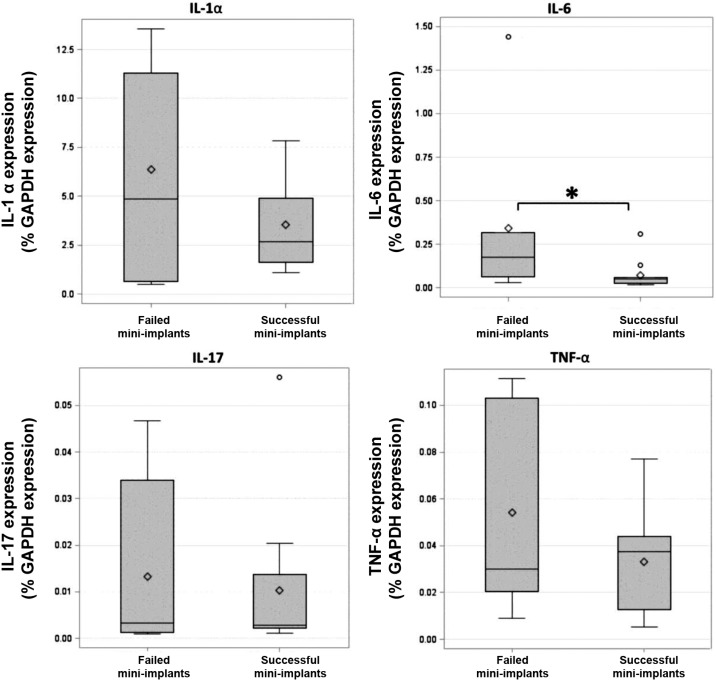
Quantification of cytokines IL-1α, IL-6, IL-17, and TNF-α, presented in box-plots as percentage of GAPDH expression, in gingival tissue around successful and failed mini-implants. * p value=0.0397. * p statistically significant for the Wilcoxon rank-sum test

The comparison between the groups relative to pro-inflammatory cytokine levels (*IL-1*α, *IL-6, IL-17*, and *TNF-*α) showed a significant difference only for *IL-6* (p=0,0397) (Figure 1), significantly higher in failed mini-implants.

**Figure 2:**
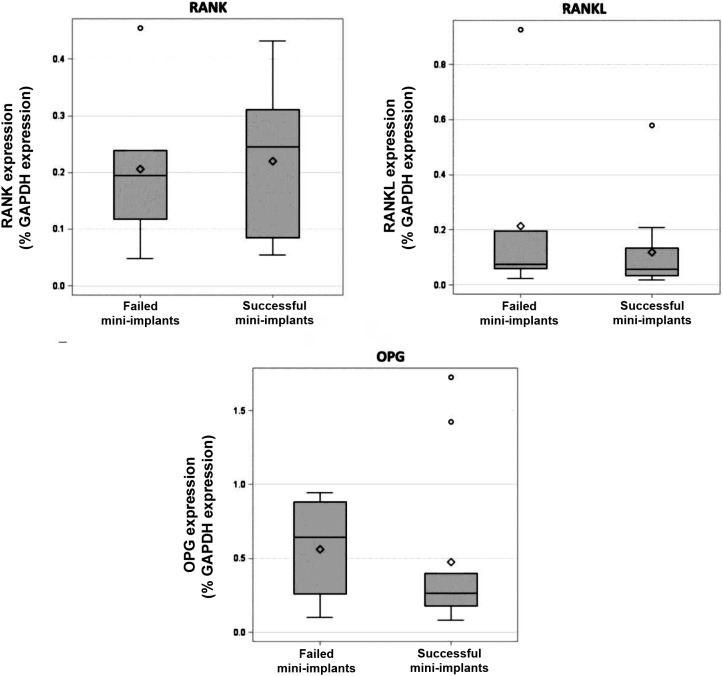
Quantification of osteoclastogenesis markers RANK, RANKL, and OPG, presented in box-plots as percentage of GAPDH expression, in gingival tissue around successful and failed mini-implants. Wilcoxon rank-sum test

Diversely, comparison between the groups revealed no statistically significant difference (p>0.05) relative to osteoclastogenesis markers (*RANK, RANKL, OPG*) (Figure 2). Medians of *RANKL/OPG* ratio were 0.2836 for successful mini-implants and 0.2278 for failed ones, without statistically significant difference between the groups (p>0.05).

## Discussion

Pro-inflammatory and anti-inflammatory cytokines regulate osteoclast and osteoblast differentiation and activation, which modulate osteoclastogenesis and maintain bone homeostasis, especially in response to aggressive agents.^6^,^7^ Cytokine overexpression involved in inflammation is critical to increase the stability of temporary anchorage devices in orthodontics.^8^ Inflammatory events are commonly identified as key elements of healing processes, inflammation mechanism remains unclear.^25^ Then, this study evaluated the gene expression of cytokines and osteoclastogenese markers to verify their role in failed mini-implants.

In this study, no significant differences were found between successful and failed mini-implants regarding *IL-1*α expression in peri-implant gingival tissue samples of both groups, but the group with failed mini-implants showed higher numerical values of this cytokine than the one with successful mini-implants. Güncü, et al.^10^ (2012) evaluated *IL-1β* expression in the peri-implant crevicular fluid of dental implants with healthy or inflamed peri-implant gingival tissues using enzyme immunoassay (ELISA) and found significantly higher *IL-1β* levels in the group with peri-implantitis. In this study, no significant differences were found between successful and failed mini-implants regarding IL-1α expression in the peri-implant gingival tissue samples of the two groups, but the group with failed mini-implants showed higher numerical values of this cytokine than the one with successful mini-implants.

Comparing both groups, difference was observed in IL-6 levels, with statistically higher levels in failed mini-implants. This result reinforces that this pro-inflammatory cytokine may play an important role in mini-implant stability loss. Severino, et al.^13^ (2011) evaluated cytokine expression in the peri-implant crevicular fluid of patients with healthy prosthetic dental implants and peri-implantitis, using enzyme immunoassay (ELISA), also finding higher IL-6 concentrations in the peri-implantitis group. Although these authors found no statistical significance, this result suggests this cytokine might interfere with local inflammation and bone resorption. Severino, et al.^13^ (2011) also reported higher IL-17 levels in patients with peri-implantitis compared with healthy dental implants. Conversely, this study revealed similar *IL-17* levels in both groups (successful and failed mini-implants). According to Sato, et al.^26^ (2006), despite acting in osteoclast activation, IL-17 also participates in neutrophil regulation, which exerts a well-defined role in periodontal infection control^27^. This could justify the statistically similar levels of this cytokine in both groups of mini-implants in this study.

TNF-α is a sensitive marker of alveolar bone loss observed in periodontitis and peri-implantitis cases. In a similar study, but with prosthetic implants, Duarte, et al.^11^ (2009) evaluated TNF-α expression in sites exhibiting different peri-implant inflammation severities using RT-PCR and found higher *TNF-*α expression in implants with severe peri-implantitis, followed by initial peri-implantitis and mucositis, which did not differ from each other. Lowest values were found in healthy sites. However, no significant difference could be found between groups regarding *TNF-*α levels in this study with orthodontic mini-implants. This difference could be due to the good use of defined groups by Duarte, et al.^11^ (2009) (implants with healthy tissues, mucositis, initial periimplantitis, and severe periimplantitis), while some degree of clinically detectable inflammation was often observed in this study, even in the successful mini-implants.

In orthodontics, RANK/RANKL/OPG system has been shown to play an important role in the bone remodeling process. In this study, no differences were observed between the groups regarding RANK, RANKL, and OPG levels. For *RANKL*, our results agree with those of Güncü, et al.^10^ (2012), who found insignificant difference in the expression of this osteoclastogenesis marker in peri-implant crevicular fluid of patients with healthy peri-implant tissues and peri-implantitis. Duarte et al.^11^ (2009) also evaluated *RANKL* levels in gingival tissue around dental implants and found *RANKL* expression was significantly lower in healthy implant sites and increased as the peri-implantitis severity increased.

Although the groups did not differ significantly regarding OPG, this cytokine levels were numerically increased in failed mini-implants compared with those of successful implants. Duarte, et al.^11^ (2009) also reported higher *OPG* expression in the group with more severe inflammation (peri-implantitis) compared with that of the group with mucositis. Güncü, et al.^10^ (2012) also found higher *OPG* levels in peri-implant crevicular fluid in patients with peri-implantitis. Lower levels were expected in this group because *OPG* acts inhibiting osteoclastogenesis, competing with *RANK* for *RANKL* binding. In this study, numerical *OPG* levels were possibly increased in failed mini-implants to control the bone resorption occurred previously.

Further studies are required to assess whether osteoclastogenesis markers expression are increased in other sites, including the interface between mini-implants and bone tissue. The role of *IL-6* in mini-implant failure should be studied using different techniques, such as immunohistochemistry, since gene expression sometimes did not reflect the protein function. The detection and immunolocalization of *RANK, RANKL* and *OPG* proteins^28^,^29^ should also be better explored, considering that the PCR used in this study detects only gene expression and not the production and localization of the proteins.

Although some authors suggest that inflammation of the mucosa adjacent to mini-implants, peri-implantitis and even bone loss mediated by the host response can be decisive for mini-implant failure,^6^,^8^,^30^ no other studies published quantified pro-inflammatory cytokines and osteoclastogenesis mediators in gingival tissue surrounding successful and failed mini-implants, hindering a direct comparison with these results.

## Conclusion

The expression of pro-inflammatory cytokines *IL-1*α, *IL-17*, and *TNF-*α and osteoclastogenesis markers *RANK, RANKL*, and *OPG* in peri-implant gingival tissue were not determinant for mini-implant stability loss. The higher expression of the *IL-6* pro-inflammatory cytokine could be associated with mini-implant stability loss.
